# Obesity attenuates the effect of sleep apnea on active TGF-ß1 levels and tumor aggressiveness in patients with melanoma

**DOI:** 10.1038/s41598-020-72481-x

**Published:** 2020-09-23

**Authors:** Carolina Cubillos-Zapata, Miguel Ángel Martínez-García, Elena Díaz-García, Ana Jaureguizar, Francisco Campos-Rodríguez, Manuel Sánchez-de-la-Torre, Eduardo Nagore, Antonio Martorell-Calatayud, Luis Hernández Blasco, Esther Pastor, Jorge Abad-Capa, Josep María Montserrat, Valentín Cabriada-Nuño, Irene Cano-Pumarega, Jaime Corral-Peñafiel, Eva Arias, Olga Mediano, María Somoza-González, Joan Dalmau-Arias, Isaac Almendros, Ramón Farré, Eduardo López-Collazo, David Gozal, Francisco García-Río

**Affiliations:** 1 Grupo de Enfermedades Respiratorias, Servicio de Neumología, Hospital Universitario La Paz-IdiPAZ, Paseo de La Castellana 261, 28046 Madrid, Spain; 2 Centro de Investigación Biomédica en Red en Enfermedades Respiratorias (CIBERES), Madrid, Spain; 3 Respiratory Department, Hospital Universitario y Politécnico La Fe, Valencia, Spain; 4 Respiratory Department, Hospital Universitario de Valme, IBIS, Seville, Spain; 5 Group of Precision Medicine in Chronic Diseases, Hospital Universitari Arnau de Vilanova and Santa Maria, IRBLleida, Lleida, Spain; 6 Dermatology Department, Instituto Valenciano de Oncología, Valencia, Spain; 7 Dermatology Department, Hospital de Manises, Valencia, Spain; 8 Respiratory Department, ISABIAL, Hospital General Universitario de Alicante, Alicante, Spain; 9 Departamento Medicina Clinica, Universidad Miguel Hernandez, Elche, Spain; 10 Respiratory Department, Hospital San Juan de Alicante, Alicante, Spain; 11 Respiratory Department, Centro de Investigacion Biomedica, Hospital Germans Trias i Pujol, Madrid, Spain; 12 Respiratory Department, Hospital Clinic- IDIBAPS, Barcelona, Spain; 13 Respiratory Department, Hospital Universitario Cruces, Bilbao, Spain; 14 Respiratory Department, Hospital Universitario Ramón y Cajal, Madrid, Spain; 15 Respiratory Department, Hospital Universitario S. Pedro Alcántara, Cáceres, Spain; 16 Respiratory Department, Hospital 12 de Octubre, Madrid, Spain; 17 Respiratory Department, Hospital Universitario de Guadalajara, Guadalajara, Spain; 18 Pneumology Department, Hospital Consorcio Terrassa, Barcelona, Spain; 19 Dermatology Department, Hospital de la Santa Creu i Sant Pau, Barcelona, Spain; 20 Unitat de Biofísica I Bioenginyeria, Facultat de Medicina i Ciències de la Salut, Universitat de Barcelona, Barcelona, Spain; 21 Institut d’Investigacions Biomèdiques August Pi i Sunyer (IDIBAPS), Barcelona, Spain; 22 TumorImmunology Laboratory IdiPAZ, Madrid, Spain; 23 Innate Immune Response Group, IdiPAZ, Madrid, Spain; 24 Department of Child Health, University of Missouri School of Medicine, Columbia, MO USA; 25 Facultad de Medicina, Universidad Autónoma de Madrid, Madrid, Spain

**Keywords:** Cancer, Diseases, Medical research, Oncology, Pathogenesis

## Abstract

Active transforming growth factor-β1 (TGF-β1), a cytokine partially regulated by hypoxia and obesity, has been related with poor prognosis in several tumors. We determine whether obstructive sleep apnea (OSA) increases serum levels of active TGF-β1 in patients with cutaneous melanoma (CM), assess their relationship with melanoma aggressiveness and analyze the factors related to TGF-β1 levels in obese and non-obese OSA patients. In a multicenter observational study, 290 patients with CM were underwent sleep studies. TGF-β1 was increased in moderate-severe OSA patients *vs.* non-OSA or mild OSA patients with CM. In OSA patients, TGF-β1 levels correlated with mitotic index, Breslow index and melanoma growth rate, and were increased in presence of ulceration or higher Clark levels. In CM patients, OSA was associated with higher TGF-β1 levels and greater melanoma aggressiveness only in non-obese subjects. An in vitro model showed that IH-induced increases of TGF-β1 expression in melanoma cells is attenuated in the presence of high leptin levels. In conclusion, TGF-β1 levels are associated with melanoma aggressiveness in CM patients and increased in moderate-severe OSA. Moreover, in non-obese patients with OSA, TGF-β1 levels correlate with OSA severity and leptin levels, whereas only associate with leptin levels in obese OSA patients.

## Introduction

The putative association between obstructive sleep apnea (OSA) and cutaneous melanoma (CM) aggressiveness^1,2^, a malignant tumor which despite substantial progress in therapy retains high mortality rates, has been reinforced by improved identification of biologically plausible pathophysiological pathways. OSA effects on tumor development and progression have been mainly attributed to the impact of intermittent hypoxia on the local tumor microenvironment, promoting melanoma cell proliferation, the release of pro-angiogenic factors, and alterations of the immune surveillance system^1,3–5^. However, the impact of OSA on the intrinsic properties of tumor cells is less well understood.

There is increasing evidence that the aggressiveness of CM is largely due to the intrinsic plasticity of melanoma cells, which is regulated by a variety of mechanisms that prominently include the transforming growth factor-β (TGF-β) pathway^6,7^. TGF-β is a multi-factorial peptide growth factor that is synthesized by macrophages, lymphocytes, fibroblasts, platelets, epithelial and cancer cells, and plays a key role in the regulation of several physiological processes, including cell proliferation, differentiation, migration, adhesion, and tissue repair^8^. TGF-β synthesized forms a dimer of with a latency-associated peptide, remaining inactive and anchored in the extracellular matrix by the TGF-ß binding proteins. TGF-ß activation requires its release form the extracellular matrix by proteolytic cleavage^9^. To adequately evaluate serum TGF-ß, it is important to consider that most of it corresponds to the latent complex form, while only a small fraction is biologically active^10^.

In melanoma cells, extracellular active TGF-β regulates gene expression by receptor-mediated activation of SMAD (an acronym from the fusion of Caenorhabditis elegans Sma genes and the Drosophila Mad, Mothers against decapentaplegic) transcription factors that regulate the transcriptional output of active genes and can also open repressive chromatin^7^. Among the several TGF-β isoforms, TGF-β1 has been shown to be expressed by melanoma cells and to promote invasiveness in cell cultures^11^, whereas attenuation of TGF-β signaling by treatment with a small molecule inhibitor reduced bone metastasis formation^12^. Although a previous study did not identify a relationship between serum TGF-ß1 concentration and melanoma stage^13^, probably because circulating bioactive TGF-β1 levels were not specifically measured, it has been demonstrated that overall canonical TGF-β/SMAD signaling is a potent promoter of melanoma progression, even at early stages of the disease^14^, and that soluble active TGF-β1 is directly related to the risk of melanoma metastasis^15,16^.

The involvement of the TGF-β/SMAD pathway in melanoma progression could be potentially important in OSA patients, since previous studies have shown that increased hypoxia-induced factor (HIF) secondary to hypoxia induces the activation of the TGF-β1/SMAD signaling pathway^17–19^. Along the same line, it has been described that patients with severe OSA have higher circulating levels of active TGF-β1 than control subjects, which decreases after six months of CPAP treatment^20^. Interestingly, it has also been reported that leptin synthesized and secreted by adipose tissues, after binding to its specific receptor on tumor cells, activates an intracellular signaling pathway that recruits transcription factors to the promoter of the TGF-β1 gene, increasing synthesis and secretion of TGF-β1 by tumor cells^21^.

Circulating TGF-β1 levels have been shown to exhibit dependency on both intermittent hypoxia and obesity (mediated through serum leptin levels)^19,21–23^, and such observations lead to the question whether OSA may have any effect on the activation of the TGF-β1/SMAD pathway in patients with melanoma, and if so, whether there is any synergistic effect with obesity. Therefore, our study objectives were to compare serum levels of active TGF-β1 in patients with CM according to the presence and severity of OSA, assess the correlation between active TGF-β1 levels and melanoma aggressiveness indices, and analyze their relationship with nocturnal hypoxia and serum levels of leptin in both obese and non-obese patients. Moreover, we explored the leptin effect on TGF-β1 released by melanoma cells under intermittent hypoxia in an in vitro model of OSA.

## Results

The general characteristics of the study participants are shown in Table 1. We included 290 patients, 146 of which were men, with a median (IQR) age of 55 (44–69) years and a mean BMI of 27.0 (24.2–29.7) Kg m^−2^. Local melanoma extension was detected in 250 patients (87%), and evidence of ulceration was found in 49 (17%). Median (IQR) Breslow and mitotic indices were 0.84 (0.50–1.93) mm and 1 (0–2) cells mm^−2^, respectively; while the growth rate was 0.15 (0.05–0.45) mm month^−1^. The overall prevalence rates of mild and moderate-to-severe OSA were 31.7 and 33.8%, respectively. The comparison between groups illustrates that, as the severity of OSA increases, patients were increasingly more likely to be men, older and more obese.

**Table 1:** General characteristics of the study subjects.

	Non OSA patients	Mild OSA patients	Moderate-severe OSA patients	*P*
N	100	92	98	-
Males, n (%)	**36 (36)**	**44 (48)**	**66 (67)**	** < 0.001**
Age (years)	**41 (36–49)**	**57 (44–73)**	**68 (59–76)**	** < 0.001**
BMI (Kg m^−2^)	**24.3 (22.6–27.2)**	**28.4 (24.9–31.5)**	**28.3 (26.6–32.3)**	** < 0.001**
Neck circumference (cm)	**36 (33–39)**	**37 (35–39)**	**40 (37–43)**	** < 0.001**
Obesity, n (%)	**7 (7)**	**27 (29)**	**37 (38)**	** < 0.001**
**Smoking status, n (%)**				0.456
Never	47 (47)	51 (55)	50 (51)	
Current smoker	23 (23)	15 (16)	14 (14)	
Past smoker	30 (30)	26 (28)	34 (35)	
**Type of melanoma, n (%)**				0.606
Superficial spreading melanoma	76 (76)	67 (73)	62 (64)	
Lentigo maligna melanoma	5 (5)	6 (7)	12 (12)	
Acral lentiginous melanoma	4 (4)	5 (5)	5 (5)	
Nodular melanoma	14 (14)	14 (15)	17 (18)	
Other	1 (1)	-	1 (1)	
Mitotic index, (cells mm^−2^)	1 (0–2.5)	1 (0–3)	1 (0–5)	0.055
Breslow index (mm)	**0.75 (0.51–1.38)**	**0.80 (0.51–2.20)**	**1.20 (0.70–3.00)**	** < 0.001**
Ulceration, n (%)	**8 (8)**	**20 (22)**	**51 (21)**	**0.014**
Growth rate, (mm month^−1^)	**0.11 (0.06–0.34)**	**0.14 (0.06–0.27)**	**0.38 (0.08–0.89)**	**0.005**
Clark index	**3 (2–3.5)**	**3 (2–4)**	**3 (3–4)**	**0.004**
**Disease stage, n (%)**				**0.048**
Localized melanoma	**92 (93)**	**80 (88)**	**78 (81)**	
Locoregional disease	**7 (7)**	**11 (12)**	**18 (19)**	
ESS score	7 (3–9)	6 (4–7)	6 (4–9)	0.353
AHI (h^−1^)	**2.0 (0.7–2.9)**	**8.6 (6.8–11.2)**	**29.8 (21.6–46.0)**	** < 0.001**
ODI (h^−1^)	**0.9 (0.3–1.4)**	**5.0 (2.6–6.8)**	**29.8 (21.6–46.0)**	** < 0.001**
Mean nocturnal SaO_2_ (%)	**94 (93–96)**	**93 (92–95)**	**93 (92–94)**	** < 0.001**
Low nocturnal SaO_2_ (%)	**89 (86–91)**	**81 (78–88)**	**79 (75–85)**	** < 0.001**
tSaO_2_ < 90% (%)	**0.0 (0.0–0.6)**	**1.7 (0.1–8.4)**	**4.5 (1.0–13.1)**	** < 0.001**
Leptin (ng mL^−1^)	3.80 (2.29–4.67)	3.60 (2.69–5.21)	3.95 (2.91–5.10)	0.131
TGF-β (pg mL^−1^)	**3.91 (2.08–7.59)**	**3.71 (0.98–9.44)**	**4.78 (2.09–11.42)**	**0.001**

Data are presented as median (interquartile range [IQR]) or n (%) and significant differences are highlighted in bold.

*BMI* body mass index; *ESS* Epworth sleepiness score; *AHI* apnea–hypopnea index; *ODI* desaturation index; *SaO*_*2*_ oxygen saturation; *tSaO*_*2*_* < 90%* night time spent with oxygen saturation < 90%; *TGF-β1* tumor growth factor- β1.

### Active TGF-β1 is increased in moderate-severe OSA patients and correlates with melanoma aggressiveness

**Figure 1:**
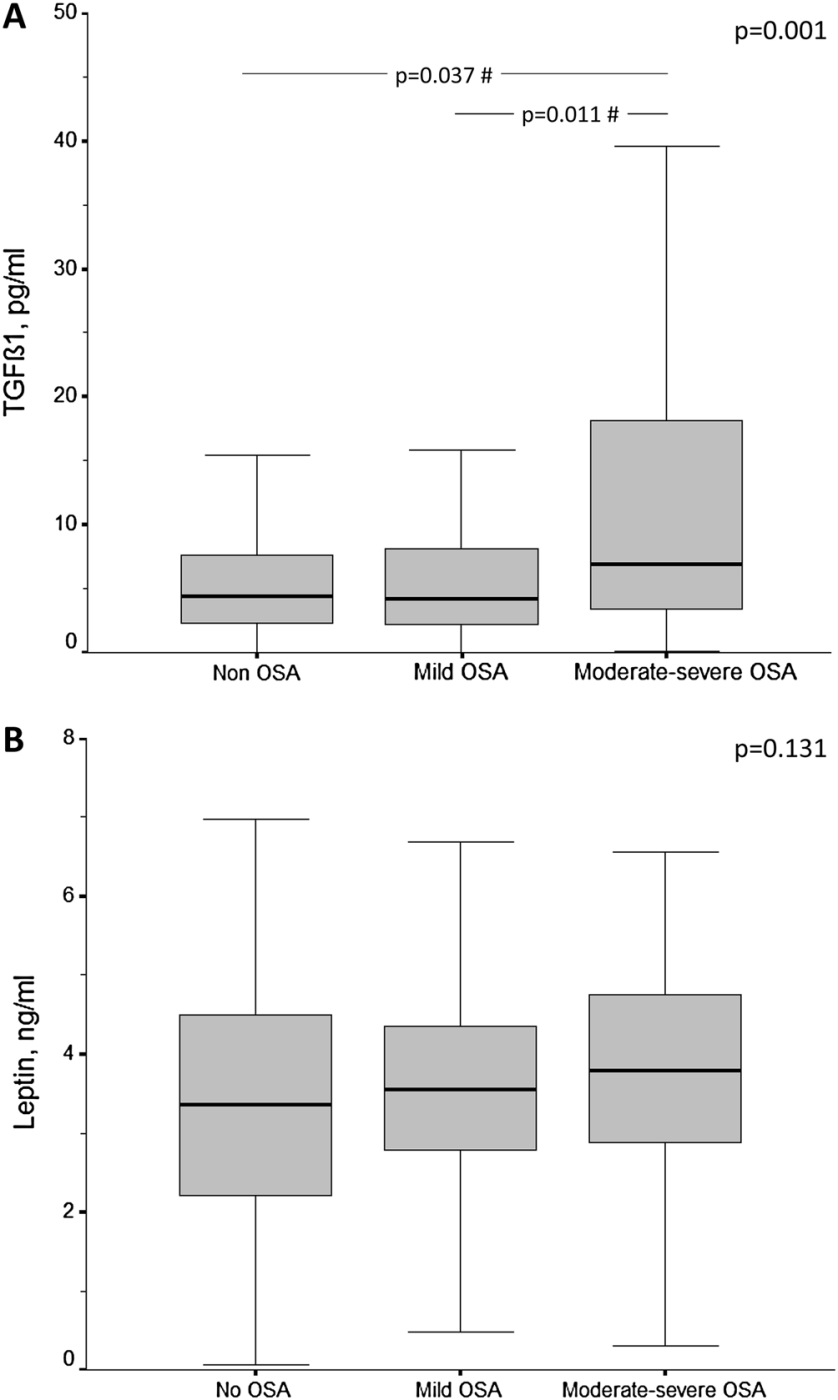
Box-and-whisker plots depicting the distribution of: (**A**) serum levels of active TGF-β1; and (**B**) leptin, in cutaneous melanoma patients according to their apnea–hypopnea index. Data are presented as median (interquartile range), maximum and minimum values, and overall comparisons were performed using the Kruskal–Wallis test. ^#^*p*-values corresponding to the post hoc comparisons between groups adjusted for sex, age, body mass index and neck circumference. *OSA* obstructive sleep apnea.

Active TGF-β1 serum levels showed significant differences among the three study sub-groups (p < 0.001). When adjusted for sex, age, BMI and neck circumference, TGF-β1 serum levels were higher in moderate-severe OSA patients versus mild OSA or non-apneic patients (Fig. 1A).

**Figure 2:**
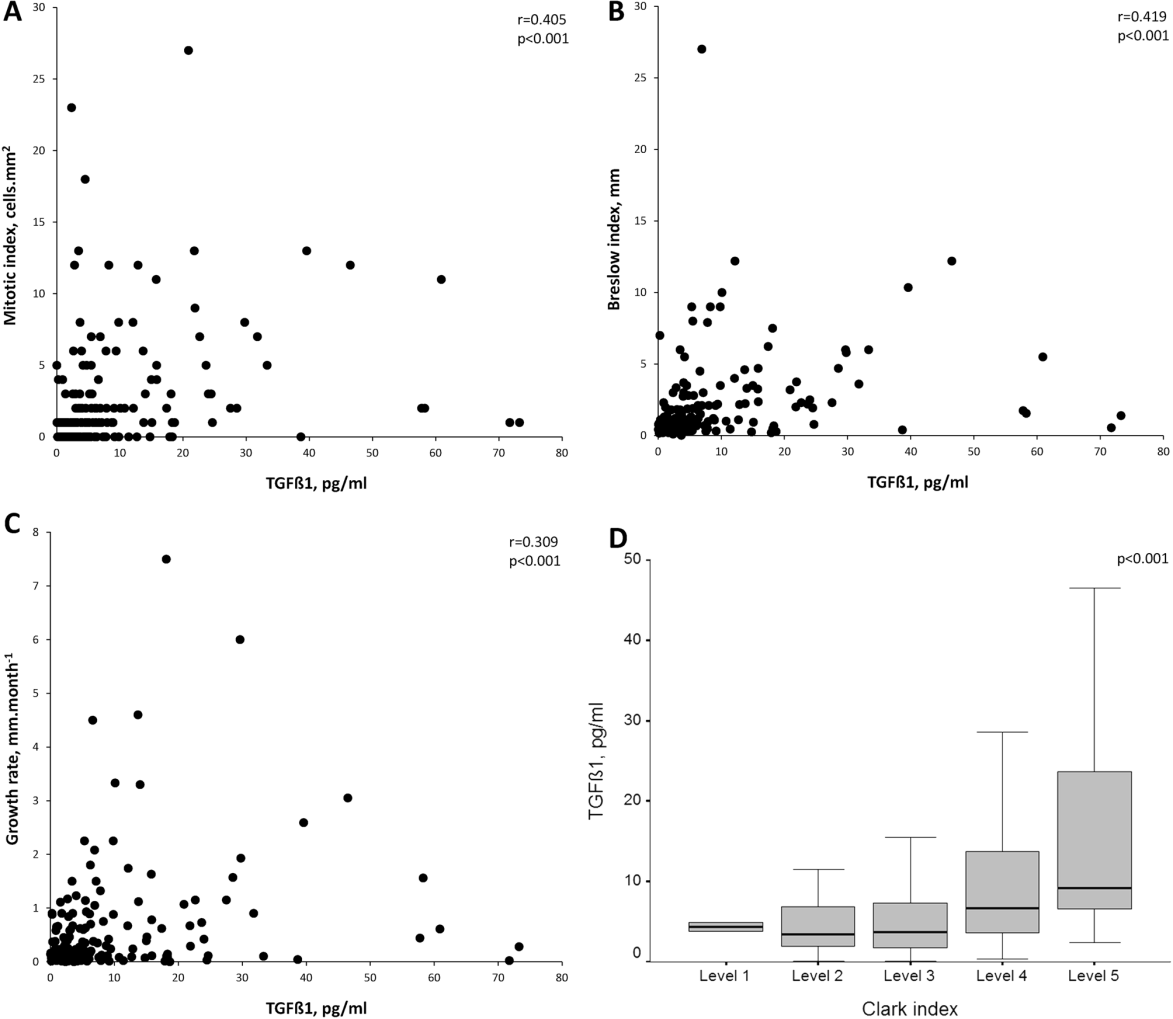
Relationship of serum levels of active TGF-β1 with the mitotic index (**A**), Breslow index (**B**) and melanoma growth rate (**C**) in patients with cutaneous melanoma and OSA. (**D**) Comparison of TGF-β1 levels according Clark level in the OSA patients with cutaneous melanoma.

In OSA patients, active TGF-β1 levels exhibited a strong relationship with mitotic index (r = 0.405, p < 0.001), Breslow index (r = 0.419, p < 0.001) and melanoma growth rate (r = 0.309, p < 0.001) (Fig. 2). OSA patients with locoregional disease had higher TGF-β1 levels than those with localized melanoma (9.64 [3.91–14.29] vs. 4.54 [2.35–9.04] pg mL^−1^, p = 0.008). TGF-β1 levels were also higher in OSA patients with ulceration of melanoma than in those without ulceration (9.07 [4.03–21.67] vs. 4.21 [2.09–8.75] pg mL^−1^, p = 0.001). Finally, differences in TGF-β1 levels according to Clark levels were also detected (p < 0.001) (Fig. 2).

### Leptin levels and OSA patient characteristics according to obesity

No significant differences in leptin levels were found among the melanoma patients without OSA and those with mild or moderate-severe OSA (Fig. 1B). In the OSA patients overall, leptin levels weakly correlated with melanoma growth rate (r = 0.192, p = 0.008) and Breslow index (r = 0.140, p = 0.054).

As would be anticipated, leptin levels were higher in obese than in non-obese OSA patients (4.49 [3.59–5.26] vs. 3.36 [2.38–4.68] ng mL^−1^, p = 0.001), whereas active TGF-β1 levels did not differ between subgroups of OSA patients (Table S1). In addition, among obese OSA patients there was a lower predominance of males as well as a greater severity of apneas-hypopneas and nocturnal hypoxemia when compared to nonobese OSA cases, but no significant differences in melanoma aggressiveness were identified.

### TGF-β1 levels only are related to OSA severity in non-obese patients

**Figure 3:**
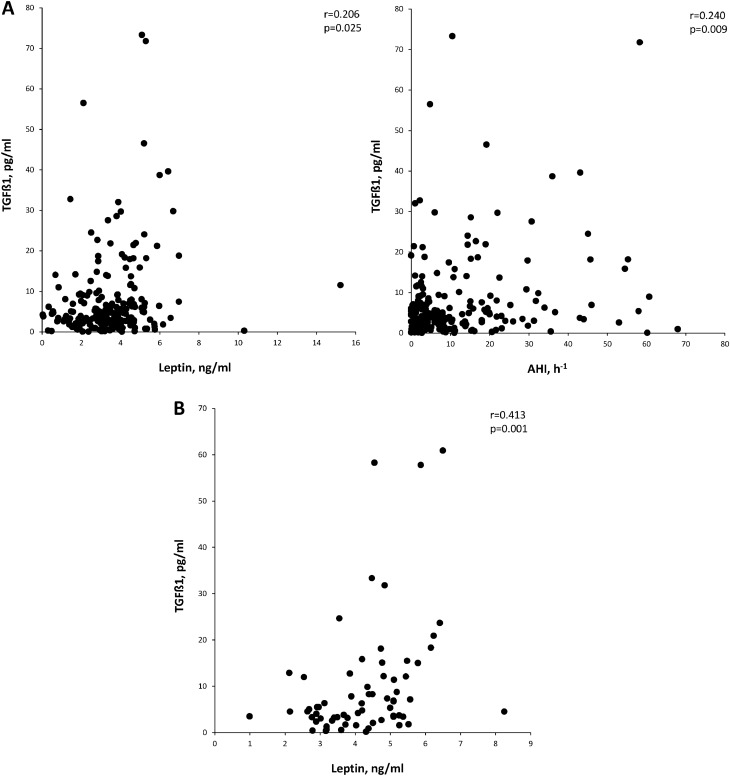
Independent factors related with serum levels of TGF-β1 in non-obese (**A**) and obese (**B**) OSA patients.

Table 2 shows the correlation between active TGF-β1 levels, polygraphic parameters and leptin levels in OSA patients with or without obesity. In non-obese OSA patients, TGF-β1 levels correlated with leptin levels as well as with AHI, desaturation index, lowest nocturnal oxygen saturation and time with SpO_2_ < 90%. In the multivariate stepwise regression model, AHI and leptin levels were retained as independent predictors of TGF-β1 levels (Table 3, Fig. 3). In contrast, in obese patients with OSA, TGF-β1 levels were only significantly related to leptin levels (Fig. 3). A sensitivity analysis using different BMI cut-off points (ranging from 28 to 33 kg m^−2^) showed similar results.

**Table 2:** Anthropometric and sleep parameters related with active TGF-β1 serum levels in the non-obese and obese OSA patients.

	Non-obese OSA patients			Obese OSA patients		
	r	95%CI	p	r	95%CI	p
Age (years)	0.013	− 0.168 to 0.193	0.887	**0.301**	**0.053 to 0.514**	**0.018**
BMI (Kg m^−2^)	0.020	− 0.161 to 0.200	0.828	− 0.029	− 0.279 to 0.224	0.825
Neck circumference (cm)	− 0.055	− 0.233 to 0.127	0.555	0.203	− 0.051 to 0.433	0.116
ESS	− 0.145	− 0.317 to 0.037	0.116	0.105	− 0.151 to 0.348	0.420
AHI (h^−1^)	**0.241**	**0.063 to 0.404**	**0.008**	0.225	− 0.028 to 0.451	0.081
DI (h^−1^)	**0.215**	**0.036 to 0.381**	**0.025**	0.059	− 0.196 to 0.306	0.651
Mean nocturnal SpO_2_ (%)	− 0.102	− 0.278 to 0.080	0.273	− 0.007	− 0.258 to 0.245	0.956
Low nocturnal SpO_2_ (%)	− **0.236**	− **0.400 to **− **0.058**	**0.014**	− 0.067	− 0.314 to 0.188	0.605
tSpO_2_ < 90% (%)	**0.208**	**0.028 to 0.375**	**0.045**	0.085	− 0.170 to 0.330	0.512
Leptin serum levels (ng mL^−1^)	**0.206**	**0.026 to 0.373**	**0.025**	**0.413**	**0.180 to 0.602**	**0.001**

*BMI* body mass index; *ESS* Epworth sleepiness score; *AHI* apnea–hypopnea index; *DI* desaturation index; *SpO*_*2*_ oxygen saturation; *tSpO*_*2*_ < *90%* night time spent with oxygen saturation < 90%; *r* Spearman correlation coefficient; *CI* confidence interval.

**Table 3:** Independent predictors of serum levels of active TGF-β1 in obese and non-obese OSA patients with melanoma.

	Unstandardized regression coefficients		95% CI for B		Standardized regression coefficients	*P* value	r^2^	r^2^ change
	B	S.E	Lower limit	Upper limit	Beta			
**Non obese OSA patients**								
Leptin level (ng mL^−1^)	2.180	0.717	0.760	3.601	0.269	0.001	0.091	0.091
AHI (h^−1^)	0.156	0.074	0.011	0.302	0.188	0.036	0.125	0.034
Constant	− 1.737	2.862	− 7.406	3.931	–	0.545	–	–
**Obese OSA patients**								
Leptin level (ng ml^−1^)	3.993	1.263	1.466	6.520	0.381	0.002	0.145	0.145
Constant	− 6.749	5.609	− 17.971	4.474	–	0.234	–	

*AHI* apnea–hypopnea index; *S.E.* standard error; *CI* confidence interval.

When all obese patients with CM were analyzed together, the presence of OSA was not associated with higher levels of TGF-β1 or with greater tumor aggressiveness. In contrast, non-obese patients with OSA exhibited higher levels of TGF-β1 and melanoma aggressiveness indices (Table S2). The correlation between serum levels of active TGF-β1 and melanoma aggressiveness indices was maintained after considering obese and non-obese OSA patients separately (Table S3).

### In vitro effect of intermittent hypoxia on TGF-β1 expression is modulated by leptin levels

**Figure 4:**
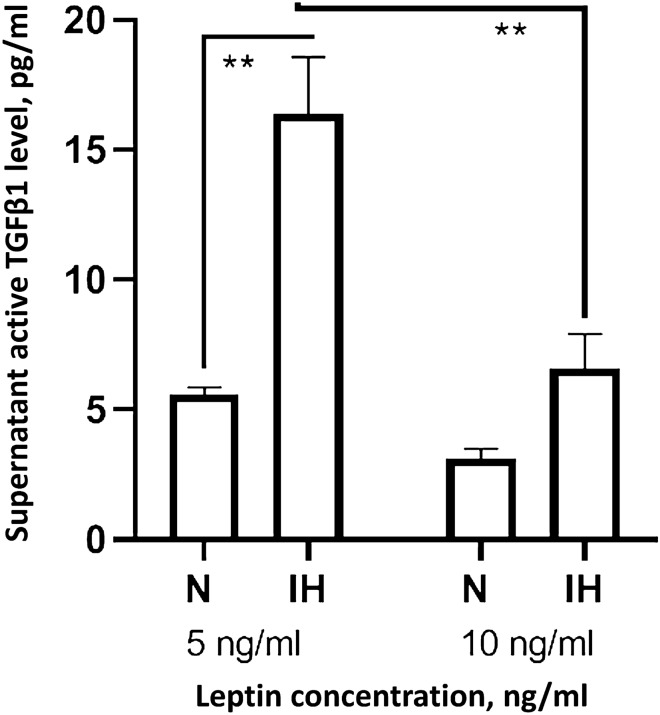
Supernatant levels of active TGF-β1 in melanoma cells treated with human leptin protein concentration at 5 ng mL^−1^ or 10 ng mL^−1^ under normoxia or intermittent hypoxia conditions (n = 7). Comparison of protein levels between groups was performed by one way ANOVA with multiple Turkey comparison. Error bars: SEM. **p < 0.01.

IH exposures in vitro increased bioactive TGF-β1 levels in the culture supernatant of melanoma cells subjected to the lowest leptin concentration, while such effect disappeared with the higher leptin concentration (Fig. 4). TGF-β1 expression assessed by mRNA showed a similar trend, although it did not reach statistical significance (Fig. S1).

## Discussion

In patients with cutaneous melanoma, moderate-severe OSA is associated with increased circulating levels of active TGF-β1 which correlate with tumor aggressiveness. In addition, while TGF-β1 levels are independently related to serum leptin and AHI in non-obese subjects, they associate with leptin levels only in obese subjects, suggesting that the potential contribution of OSA to increase active TGF-β1 may be limited to non-obese patients. Moreover, the in vitro melanoma cell culture model showed that the IH-induced increase of active TGF-β1 expression is attenuated by the presence of higher leptin levels.

The TGF-β pathway plays a major role in the evolution of cancer, and has been implicated with tumor progression by driving epithelial mesenchymal transition, tumor cell migration, invasion, metastatic spread and angiogenesis^24^. The roles of TGF-β1 have been more extensively investigated relative to other isoforms of TGF-β. When bound to a specific receptor on the target cell membrane, TGF-β1 activates TGF-β receptor type I kinase, resulting in the phosphorylation of SMAD2 and SMAD3, which subsequently form oligomeric complexes with SMAD4, and translocate to the nucleus to regulate the expression of a large repertoire of genes^21,25^. In light of the biological roles of TGF-β1 in cancer, the strong relationships between serum levels of active TGF-β1 and all indices of melanoma aggressiveness in OSA patients are therefore not unexpected. The increased TGF-β1 levels identified in patients with moderate-severe OSA indicate that this disorder has an incremental effect on the expression and release of active TGF-β1 even in patients who have already developed a melanoma. Currently available evidence suggests that this effect is likely due to the OSA-related intermittent hypoxia. In fact, TGF-β1 has been shown to be increased in a variety of cells cultured in hypoxia and also in hypoxic ischemic tissues^26^. Several reports show that hypoxia activates latent TGF-β1 via HIF-1α in hepatocytes, placental fibroblasts, primary human lung fibroblasts, smooth muscle cells and cancer cells^22,26^. Using in vitro models, it has been recently confirmed that HIF-1α induction by hypoxia promotes the expression of TGF-β1, activates its receptors, and increases the phosphorylation of the SMAD2/3 proteins, indicating an activation of the TGF-β/SMAD signaling pathway, probably through the TLR/MyD88/NF-κB pathway^23^.

Although our results do not establish causal inferential mechanisms, the correlation found between the different nocturnal hypoxemia indices and the levels of active TGF-β1 among patients with OSA and melanoma concurs with the potential relevance of intermittent hypoxia in the enhanced induction of TGF-β1. The selection of AHI instead of the nocturnal hypoxemia indices by the multiple regression model could be due solely to the limited number of patients with more severe OSA (cutaneous melanoma was the driving selection criterion rather than OSA). However, it is also not possible to exclude that other OSA-related perturbations contribute to TGF-β1 production. Indeed, in a murine model exposed to intense sleep fragmentation, increases in TGF-β1 expression in the hypothalamus and hippocampus were reported^27^.

Leptin is primarily produced by fat cells and regulates food intake, energy homeostasis and cell proliferation^28^. High serum levels of leptin, which are strongly correlated with adipose tissue mass, are recognized as a factor driving cancer development and progression, as leptin exhibits mitogenic, proinflammatory, anti-apoptotic, and proangiogenic properties^28–31^. The finding of a significant relationship between leptin and TGF-β1 levels in our patients is consistent with previous studies that showed that leptin binds to leptin receptors on the surface of cancer cells, activates JAK/STAT3 signaling, and consequently increases TGF-β expression^21^. Moreover, it has been demonstrated that leptin can be a co-factor for TGF-β in cancer using an in vitro model of kidney fibroblasts whereby leptin treatment enhances SMAD2/3 phosphorylation by TGF-β^32,33^. Interestingly, the relationship between leptin and TGFβ could be bidirectional, since TGFβ/SMAD3 signaling appears to be crucial for the development of obesity, as demonstrated in mice in which TGFβ signaling was blocked or that were deficient in SMAD3 and consequently were protected from developing obesity^34,35^. Additionally, other members of the TGF-β family are involved in regulation of adipogenesis and energy metabolism through SMAD and P38 signaling pathways^36^.

There is substantial evidence on the association between obesity and increased cancer risk and metastasis^37^. It has been estimated that roughly 20% of all cancers are caused by excess weight gain^38,39^ and, although the mechanisms are unclear, some studies have suggested that obesity enhances metastases in tumors such as melanoma, colon, lung and breast cancer^40,41^. In fact, there is greater risk of recurrence among cancer patients with higher BMI^42^, and cancer patients with high BMI have higher mortality rates compared to those with average BMI^38^. Although in our subgroup of patients with OSA and melanoma the differences in Breslow index between obese and non-obese subjects did not reach statistical significance (Table S1) (probably due to sample size), in the overall cohort, those who were obese had a higher Breslow index compared to non-obese (1.10 [0.60–2.15] vs. 0.80 [0.47–1.70] mm, p = 0.015), further reinforcing the observation that obesity is associated with greater melanoma aggressiveness.

**Figure 5:**
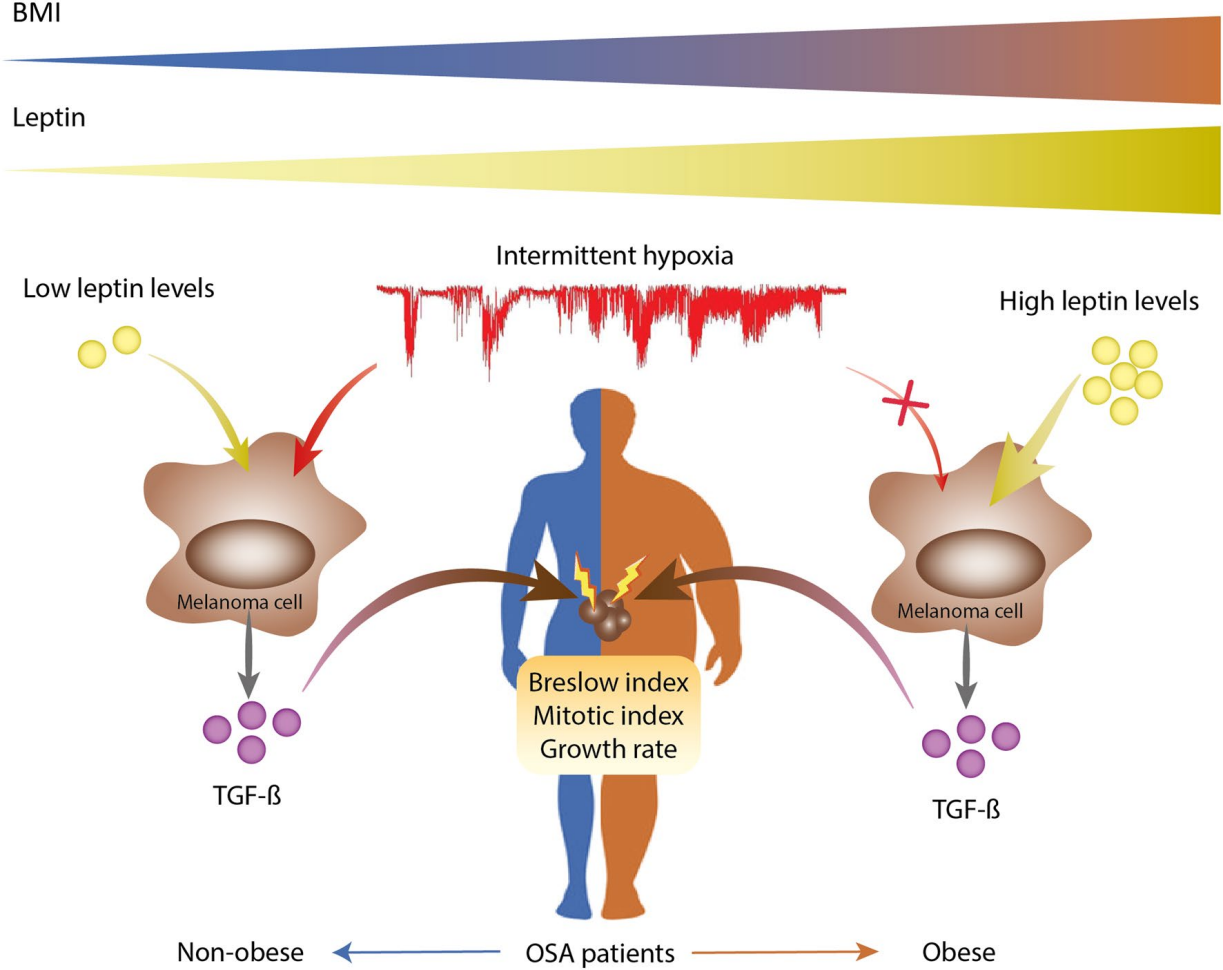
Schematic representation of the proposed interaction between intermittent hypoxia, obesity, and circulating levels of TGF-ß in patients with melanoma and OSA. In non-obese subjects, OSA-induced intermittent hypoxia could have a synergistic effect with leptin produced by adipocytes on the TGF-ß expression by melanoma cells, promoting greater tumor aggressiveness. According to the data of the present study, this effect is lost in obese patients, since the basal overexpression of TGF-ß caused by high levels of leptin is not enhanced by the additional presence of intermittent hypoxia.

The most striking finding in the present study is that the correlation between AHI and nocturnal hypoxemia indices with the serum levels of active TGF-β1 among OSA patients reached statistical significance only in non-obese subjects, while in the obese the serum leptin concentration was the only independent variable associated with TGF-β1 levels. Moreover, the results of our in vitro model confirmed that intermittent hypoxia-induced elevations in active TGF-β1 expression in melanoma cells are attenuated by higher leptin levels. In this context, our results suggest that the potential contribution of OSA to melanoma aggressiveness is limited to non-obese patients (Fig. 5). This finding concurs with the results from a murine subcutaneous melanoma model, in which the application of intermittent hypoxia mimicking OSA increased the growth of melanoma tumors in lean mice, but not in obese mice^43^. In fact, the increased tumor growth induced by obesity was not enhanced by adding the intermittent hypoxia stimulus, and the effect of intermittent hypoxia on tumor necrosis, and vascular density was dampened in the context of obesity^43^. Likewise, a previous analysis of the entire cohort of melanoma patients used in the present study showed that the probability of having a Breslow index > 1 mm in patients with an AHI or a desaturation index in the upper tertile compared with the reference group was higher in non-obese than in obese patients^1^. Although our results do not provide a mechanistic explanation for this finding, we speculate that the lack of a relationship between sleep parameters and active TGF-β1 levels in obese patients with OSA might be attributable to the obesity-induced activation of the NFkB pathway, which could attenuate the OSA-induced HIF-1α -TGF-β production pathway. On the other hand, we also cannot rule out competition for binding at specific sites of the TGF-β1 regulatory gene between transcription factors induced by the activation of the leptin receptor and by the increase of HIF-1α.

In addition to its multicenter character and the relatively large cohort size, as well as the homogenization and rigorous control of sleep studies performed by the Spanish Sleep Network, we believe that the main strength of this study was the measurement of free active TGF-β1 in serum, which more reliably reflects effector activity of its several biological functions, such as activating epithelial mesenchymal transition, proliferation and the spread of metastasis^44^. However, our study has several limitations that must be acknowledged. First, the sleep study was performed using respiratory polygraphy rather than polysomnography, and it is therefore not possible to evaluate the contribution of sleep fragmentation. Second, there was a certain delay between the skin biopsy and polygraphic studies (which was not different between the study groups and never exceeded 4 months). Although no changes in patient weight or any other potential confounding factors were identified, we cannot rule out that such factors were void of any influence on our results. Third, as in most studies recruiting patients who had not been referred to sleep units for suspicion of OSA, daytime sleepiness in the OSA groups is low, which may be a limitation to extrapolate our findings to much more symptomatic patients. Fourth, we did not have a group of patients with distant metastatic melanoma to evaluate their serum TGF-β1 and leptin levels. Fifth, our study does not provide any information on the effect of OSA treatment on TGF-β1 levels or its effect on melanoma aggressiveness, and therefore no therapeutic recommendations beyond those in place for OSA can be formulated.

In summary, our study shows that melanoma patients with moderate-severe OSA have higher serum levels of active TGF-β1, which are directly related to melanoma aggressiveness. Furthermore, in non-obese patients with OSA TGF-β1 level variance is dependent on both AHI and serum leptin levels, while in obese patients with OSA only leptin levels retain their independent association with TGF-β1 levels.

## Methods

### Study subjects

Adult patients with new diagnoses of cutaneous melanoma were consecutively selected from a multicenter, observational study involving 29 teaching hospitals in Spain^1^. Exclusion criteria included extra-cutaneous location of the melanoma, daytime respiratory or heart failure, and current or previous use of home oxygen therapy, continuous positive airway pressure (CPAP) or noninvasive mechanical ventilation. Out of 443 recruited patients, serum samples were obtained in 360 subjects and after their clinical use, were available in 290 patients. The study was approved by the Institutional Ethics Committee of the Hospital Universitario y Politécnico La Fe, Valencia, Spain (2016/0223), and all subjects gave their written informed consent. All the experiment protocol for involving humans was in accordance to Declaration of Helsinki.

### Dermatological evaluation

As previously described^45,46^, patients were evaluated by a dermatologist at each hospital, and data for tumor location and clinical stage at diagnosis (categorized as localized or loco-regional disease) were registered. All tumors were surgically removed, and the Breslow tumor thickness, ulceration, tumor mitotic rate, and Clark levels were calculated^47^. Melanoma growth rate was calculated as the Breslow index divided by the difference between the date of the tumor excision and the date on which patients noticed changes suggestive of malignant transformation in a stable, pre-existing lesion or the time when they noticed the appearance of a new and changing lesion^46^. All dermatological and pathological evaluations were blinded to the sleep study and biochemical analysis findings.

### Sleep study

Anthropometric characteristics were measured and, according to WHO criteria, obesity was defined as a body mass index (BMI) ≥ 30 kg m^−2^. Simultaneously, all patients underwent overnight respiratory polygraphy within a maximum of 4 months after CM diagnosis. All scoring and readings were conducted manually by experienced and trained personnel. Apnea was defined as an interruption of oronasal flow of > 10 s. Hypopnea was defined as a 30–90% reduction in the oronasal airflow for > 10 s associated with an oxygen desaturation ≥ 3%. The apnea–hypopnea index (AHI) was defined as the number of apneas plus hypopneas per hour of recording, while tSat90 was defined as the percentage of recording time with SaO_2_ < 90%. In addition, mean saturation, minimum saturation and oxygen desaturation index (ODI) were measured. According to the AHI, patients were divided into three groups: non-OSA (AHI 0–5 h^−1^), mild OSA (AHI 5–15 h^−1^) and moderate-severe OSA (AHI > 15 h^−1^)^48^.

### Determination of serum levels of active TGFβ1 and leptin

Fasting venous blood samples were drawn between 8 and 9 am. The blood samples were centrifuged to separate the serum, and all specimens were immediately aliquoted, frozen and stored at − 80 °C. Free active TGF-β1 and leptin in serum were assayed using human free active TGF-β1 and leptin enzyme linked immunosorbent assay (437707 Legend Max, San Diego, USA and MBS9501873 MyBiosource, San Diego, USA, respectively) according to the manufacturers’ instructions. The assays were done in duplicates in all samples. The detection limits of the assays were 63 pg mL^−1^ and 2.3 pg mL^−1^, respectively. The intra-assay and inter-assay variations were below 10% in all cases.

### Cell culture

C81-61 human cutaneous melanoma cell line^49^ was cultured under normoxia or intermittent hypoxia (IH) conditions over night, as previously described^50^. The culture medium was supplemented with human leptin (230-30112-10, RayBiotech) at either a concentration similar to that detected in patients or two-fold that level (5 and 10 ng mL^−1^, respectively). Supernatant concentrations of active TGF-β1 were measured by ELISA as in serum.

### RNA isolation, quantification, and analysis

Total RNA was purified using the High Pure RNA Isolation Kit (Ambion, Carlsbad, CA, USA). cDNA was obtained using the High Capacity cDNA Reverse Transcription Kit (Applied Biosystems). Gene expression levels were analyzed by real-time quantitative PCR using the LightCycler System (Roche Diagnostics, Basel Switzerland); and quantitative PCR was performed using a QuantiMix Easy SYG Kit (Biotools; Madrid, Spain). The results were normalized to the expression of 18S, and the cDNA copy number of each gene of interest was determined using a 7-point standard curve. The products were amplified using specific primers as described previously^20^. The products were amplified using primers for TGF-β1 5′-GGCCAGATCCTGTCCAAGC-3′ (forward) and 5′- GTGGGTTTCCACCATTAGCAC-3′ (reverse).

### Statistical analysis

According the type and distribution of the variables, data are expressed as mean ± SD, median (interquartile range [IQR]) or absolute numbers and percentages. Normality was explored using the Shapiro–Wilk and skewness-kurtosis tests. Differences between groups were analyzed using the Chi-squared or Fisher’s exact tests (categorical variables) and Kruskal–Wallis or Mann–Whitney U tests (ordinary or non-normal metric variables). For between-group comparisons, TGF-β1 and leptin levels were adjusted for gender, age, body mass index and neck circumference using general linear models. Correlations between parameters were evaluated using Spearman’s correlation. Those variables exhibiting statistically significant findings were then introduced into a multiple linear regression analysis to identify independent determinants of TGF-β1 levels. Stepwise methods were used to include or remove individual independent variables at each step, based on the probability of F (entry: 0.05; removal: 0.10). Other aspects explored included residual standard deviation, changes in the distribution of the residuals and the homogeneity of the variance over the predictors^51^. Analyses were performed using the Statistical Package for the Social Sciences (version 20.0; SPSS, Chicago, IL, USA).

## Supplementary information

Supplementary file1
