# Supplementary material for: Obesity attenuates the effect of sleep apnea on active TGF-ß1 levels and tumor aggressiveness in patients with melanoma
Source: Sci Rep. 2020 Sep 23;10:15528. doi: 10.1038/s41598-020-72481-x (PMC7511355; doi:10.1038/s41598-020-72481-x)

Supplementary information

Title: **Obesity attenuates the effect of sleep apnea on active TGF-ß1 levels and tumor aggressiveness in patients with melanoma**

Authors: Carolina Cubillos-Zapata^1,2^, Miguel Ángel Martínez-García^3^, Elena Díaz-García^1,2^, Ana Jaureguizar^1^, Francisco Campos-Rodríguez^2,4^, Manuel Sánchez-de-la-Torre^2,5^, Eduardo Nagore^6^, Antonio Martorell-Calatayud^7^, Luis Hernández Blasco^8,9^, Esther Pastor^10^, Jorge Abad-Capa^2,11^, Josep María Montserrat^2,12^, Valentín Cabriada-Nuño^13^, Irene Cano-Pumarega^14^, Jaime Corral-Peñafiel^2,15^, Eva Arias^16^, Olga Mediano^2,17^, María Somoza-González^18^, Joan Dalmau-Arias^19^, Isaac Almendros^2,20,21^, Ramón Farré^2,20,21^, Eduardo López-Collazo^2,22,23^, David Gozal^24^, and Francisco García-Río^1,2,25^

**Table S1**. Characteristics of OSA patients with melanoma according presence or absence of obesity*

|  | | **Obese patients with OSA** | **Non-obese patients with OSA** | **p** |
| --- | --- | --- | --- | --- |
| N | | 64 | 126 | - |
| **Males** | | **30 (47)** | **80 (64)** | **0.021** |
| Age, yr | | 65 (53-72) | 68 (53-76) | 0.845 |
| **BMI, Kg/m^2^** | | **33.0 (31.4-35.7)** | **26.9 (24.6-28.3)** | **<0.001** |
| **Neck circumference, cm** | | **41 (38-45)** | **38 (37-41)** | **<0.001** |
| Smoking status | | | | 0.807 |
|  | Never | 32 (50) | 69 (55) |  |
|  | Current smoker | 10 (17) | 19 (15) |  |
|  | Past smoker | 22 (34) | 38 (30) |  |
| Mitotic index, cells.mm^-2^ | | 1.0 (0.0-4.3) | 1.0 (0.0-5.0) | 0.575 |
| Breslow index, mm | | 1.16 (0.76-3.00) | 1.00 (0.51-2.35) | 0.071 |
| Ulceration, n (%) | | 11 (17) | 30 (24) | 0.195 |
| Growth rate, mm.month^-1^ | | 0.20 (0.08-0.68) | 0.22 (0.08-0.88) | 0.226 |
| Clark index | | 4 (3-4) | 3 (3-4) | 0.140 |
| ESS score | | 6 (3-9) | 6 (4-9) | 0.156 |
| **AHI, h^-1^** | | **24.4 (10.8-50.6)** | **19.7 (11.0-31.4)** | **0.017** |
| **ODI-4, h^-1^** | | **14.7 (6.1-34.3)** | **11.5 (5.6-19.7)** | **0.011** |
| **ODI-3, h^-1^** | | **24.6 (12.5-40.3)** | **18.2 (12.0-30.4)** | **0.017** |
| **Mean nocturnal SaO_2_, %** | | **93 (91-93)** | **94 (93-95)** | **<0.001** |
| **Low nocturnal SaO_2_, %** | | **79 (75-84)** | **82 (76-86)** | **0.012** |
| **tSaO_2_ <90%, %** | | **5.4 (1.4-21.6)** | **2.7 (0.4-6.8)** | **0.002** |
| **Leptin, ng/ml** | | **4.49 (3.59-5.26)** | **3.36 (2.38-4.68)** | **0.001** |
| TGF-β1, pg/ml | | 6.5 (2.6-15.2) | 3.9 (1.0-8.2) | 0.326 |

*Data are presented as median (interquartile range [IQR]) or n (%).

Abbreviations: BMI, body mass index; ESS, Epworth sleepiness score; AHI, apnea-hypopnea index; ODI, desaturation index; SaO_2_, oxygen saturation; tSaO_2_<90% night time spent with oxygen saturation < 90%; TGF-β1, tumor growth factor-ß1.

**Table S2**. Melanoma aggressiveness indices and TGF-β1 and leptin levels in non-obese and obese patients according OSA severity*

|  | | **Non OSA** | **Mild OSA** | **Moderate-severe OSA** | ***P* value** |
| --- | --- | --- | --- | --- | --- |
| TGF-β1 serum level, ng/ml | | | | | |
|  | Non-obese subjects | **4.13 (2.17-7.26)** | **3.68 (2.09-5.56)** | **6.14 (2.93-16.91)** | **0.020** |
|  | Obese subjects | 7.58 (2.99-12.86) | 3.96 (1.49-7.60) | 6.73 (3.35-15.07) | 0.154 |
| Leptin serum level, pg/ml | | | | | |
|  | Non-obese subjects | 3.34 (2.13-4.46) | 3.50 (2.58-4.04) | 3.46 (2.76-4.72) | 0.358 |
|  | Obese subjects | 4.79 (3.43-5.19) | 4.35 (3.19-5.28) | 4.35 (3.26-5.10) | 0.561 |
| Locoregional disease, n (%) | | | | | |
|  | Non-obese subjects | 7 (8) | 8 (12) | 12 (20) | 0.077 |
|  | Obese subjects | 0 | 3 (12) | 6 (17) | 0.468 |
| Mitotic index, cells.mm^-2^ | | | | | |
|  | Non-obese subjects | **1 (0-2)** | **1 (0-2)** | **1 (0-4)** | **0.030** |
|  | Obese subjects | 2 (0-3) | 1 (0-2) | 1 (0-5) | 0.857 |
| Breslow index, mm | | | | | |
|  | Non-obese subjects | **0.72 (0.48-1.13)** | **0.70 (0.40-2.03)** | **1.10 (0.56-2.34)** | **0.004** |
|  | Obese subjects | 1.05 (0.66-2.20) | 0.94 (0.70-2.23) | 1.75 (0.75-3.50) | 0.523 |
| Ulceration, n (%) | | | | | |
|  | Non-obese subjects | **7 (8)** | **15 (23)** | **15 (25)** | **0.006** |
|  | Obese subjects | 1 (14) | 5 (19) | 6 (16) | 0.953 |
| Growth rate, mm-month^-1^ | | | | | |
|  | Non-obese subjects | **0.11 (0.05-0.31)** | **0.17 (0.05-0.33)** | **0.22 (0.05-0.90)** | **0.046** |
|  | Obese subjects | 0.13 (0.08-0.27) | 0.20 (0.08-0.66) | 0.39 (0.10-0.89) | 0.359 |
| Clark index | | | | | |
|  | Non-obese subjects | **3 (2-3)** | **3 (2-3)** | **3 (3-4)** | **0.020** |
|  | Obese subjects | 3 (2-3) | 3 (2-4) | 4 (2-4) | 0.511 |

*Data are presented as median (interquartile range [IQR]) or n (%).

Abbreviations: TGF-β1, tumor growth factor-ß1.

**Table S3**. Relationship of the serum levels of active TGF-β1 with the melanoma aggressiveness indices in OSA patients with or without obesity *

|  | **Non-obese OSA patients** | | |  | **Obese OSA patients** | | |
| --- | --- | --- | --- | --- | --- | --- | --- |
|  | **r** | **95%CI** | ***P*** |  | **r** | **95%CI** | ***P*** |
| Mitotic index, cells.mm^-2^ | 0.370 | 0.209 to 0.512 | <0.001 |  | 0.479 | 0.264 to 0.648 | <0.001 |
| Breslow index, mm | 0.403 | 0.245 to 0.540 | <0.001 |  | 0.419 | 0.193 to 0.603 | 0.001 |
| Growth rate, mm.month^-1^ | 0.277 | 0.107 to 0.431 | 0.002 |  | 0.348 | 0.112 to 0.547 | 0.001 |
| Clark index | 0.401 | 0.243 to 0.538 | <0.001 |  | 0.392 | 0.162 to 0.582 | 0.001 |

*Abbreviations: CI, confidence interval.

**Figure S1**. *In vitro* TGF-β1 quantification assays. The melanoma cell lines were treated with human leptin protein concentration at 5 and 10 ng/mL for 16 hours under normoxia conditions (n=7). Then, the cell cultures were split into normoxia or intermittent hypoxia conditions over night. The TGF-β1 mRNA expression analyses were evaluated by qPCR in melanoma treated cell culture. Comparisons between groups were performed by one-way ANOVA with multiple Tukey comparison. Error bars: SEM.


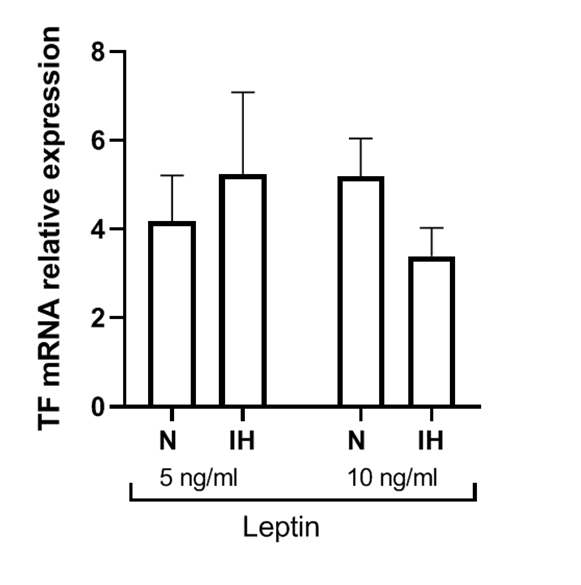

Supplement: Supplementary file 1 — Supplementary file1 [file 41598_2020_72481_MOESM1_ESM.docx]
